# Towards Translational Epidemiology: Next-Generation Sequencing and Phylogeography as Epidemiological Mainstays

**DOI:** 10.1128/mSystems.00119-19

**Published:** 2019-06-11

**Authors:** Crystal M. Hepp

**Affiliations:** a School of Informatics, Computing, and Cyber Systems, Northern Arizona University, Flagstaff, Arizona, USA; b The Pathogen and Microbiome Institute, Northern Arizona University, Flagstaff, Arizona, USA

**Keywords:** public health, translational epidemiology, vector-borne diseases, zoonotic disease

## Abstract

Next-generation sequencing, coupled with the development of user-friendly software, has achieved a level of accessibility that is revolutionizing the way we approach epidemiological investigations. We can sequence pathogen genomes and conduct phylogenetic analyses to assess transmission, identify from which country or city a pathogen originated, or which contaminated potluck item resulted in widespread foodborne illness. However, until recently, these types of studies have been rarities, limited to specific investigations usually conducted over the short term. Given the feasibility and realized public health benefits of ascertaining pathogen relationships, federal, state, and county agencies are building their sequencing capacities, either through acquisition of equipment or collaborative activities. In this perspective, I detail research projects that our group collaborates on with county and state public health agencies, where the objective is to identify pathogen source locations with the longer-term goal of implementing proactive interventions.

## Abstract (precis)

Next-generation sequencing, coupled with the development of user-friendly software, has achieved a level of accessibility that is revolutionizing the way we approach epidemiological investigations. We can sequence pathogen genomes and conduct phylogenetic analyses to assess transmission, identify from which country or city a pathogen originated, or which contaminated potluck item resulted in widespread foodborne illness.

## PERSPECTIVE

The identification of West Nile virus (WNV) in the United States in 1999 stimulated vector-borne zoonotic disease surveillance through appropriated federal funding disseminated nationwide (1). For many vector control agencies, this funding allowed for the development of large-scale search and destroy programs, where high vector prevalence or arbovirus-positive samples collected from traps trigger reactive intervention measures. Additionally, surveillance data are relayed back to federal, state, and county public health agencies at higher geographic scales through ArboNET (2), which provides county level resolution of reportable arboviral presence in mosquito pools, humans, and animals. While regional presence and relative prevalence of pathogens has been leveraged to predict human risk (3), little can be ascertained from these traditional practices in the way of disease spread or reemergence. However, vector-borne and other zoonotic infectious disease epidemiology at the agency level is approaching a paradigm shift, largely propelled by the advent and feasibility of next-generation sequencing, innovative and user-friendly phylogenomic software packages, and motivation of the One Health Movement. Briefly, the One Health concept is a realization that human, animal, and environmental health are inextricably intertwined, largely due to overlapping habitats, activities, food sources, etc. Approaches to address One Health-related outbreaks require meticulous record-keeping and cooperation among stakeholders at all levels, from federal agencies to small farm owners, which is often challenging. A recent national investigation of the largest Escherichia coli O157:H7 outbreak since 2006, resulting in 210 cases and five deaths, demonstrated the ability of a One Health approach to locate pathogen sources of human illness (4). The Food and Drug Administration, Centers for Disease Control and Prevention (CDC), and state partners worked with leafy greens farms, processing facilities, cattle feeding operations, and water districts to conduct an investigation that would ultimately identify a regional source of contaminated romaine lettuce. Of the numerous samples collected, only three samples from an irrigation canal were positive for the outbreak strain, as determined by whole-genome sequencing. While a direct link could not be made to environmental contamination by animals, a large cattle feeding operation is located along the sampled segment of the canal, and canal water was applied to some lettuce crops. This investigation is an exemplary model for One Health approaches moving forward, and with the CDC making recent technological investments in public health laboratories as part of the Advanced Molecular Detection Initiative, we can expect that these investigations will form epidemiological foundations.

In addition to sequencing accessibility, the development of user-friendly software that allows for the incorporation of geographic coordinates, timing information, and other metadata (e.g., BEAST [5]) has paved the way for better understanding regional circulation of infectious diseases, identifying probable source locations and reservoirs from which these diseases emerge, and factors that encourage or mitigate spread. Such methods have been recently employed to understand the circulation of Ebola virus during the 2013–2016 epidemic in West Africa (6) and Zika virus in the 2015–2016 epidemic in the Americas (7). In collaboration with county vector control agencies, state public health departments, the Pathogen and Microbiome Institute at Northern Arizona University, and the Pathogen Genomics Division of the Translational Genomics Research Institute, our research team employs the described technological approaches within a One Health framework toward characterizing the circulation of three annually reemerging zoonotic viruses, at various geographic scales, to identify locations that are critical in long-term local maintenance.

## WEST NILE VIRUS

WNV was first identified in New York City in 1999 and successfully migrated across the continental United States by 2004. Nearly 2 decades after the initial introduction, WNV is still the most important arbovirus nationwide, causing 95% of arboviral diseases reported to the Centers for Disease Control and Prevention (CDC) (8). While WNV has been variably present in counties across the United States from year to year, positive mosquito pools and human clinical cases have occurred in Maricopa County in Arizona every year after the first detection in September 2003. In response, the Maricopa County Environmental Services Vector Control Division has developed what is likely one of the most intricate arbovirus surveillance systems in the United States. With more than 800 carbon dioxide traps distributed throughout the Phoenix metropolitan area, collecting mosquitoes that are tested for arboviral activity each week, the county consistently detects the environmental threat of WNV each year prior to the occurrence of human clinical cases—the hallmark of an effective surveillance system.

We have initiated an extensive collaboration with the county, which involves sequencing WNV genomes from all positive mosquito pools they collect each year, layered with geographic and timing information, to better understand whether WNV is maintained in Maricopa County each year or is annually imported, and to identify source locations. Our efforts, which now include more than 300 sequenced genomes from 2014 to 2018, have shown that the currently circulating population of WNV has been endemic in Maricopa County since 2013 (9). We have additionally incorporated other southwestern locations, and a preliminary analysis resulting from our collaborations with Yuma County (Arizona) Pest Abatement District and Coachella (California) Valley Mosquito and Vector Control District has revealed that the endemic population of WNV in Maricopa County, at least during 2017, acted as a source for WNV strains that emerged and thrived in Yuma County, AZ, and Riverside County, CA. We just received samples from these locations for 2018, and we are in the process of investigating whether or not the same patterns reemerge, which would suggest that Maricopa County is an important source location for WNV maintenance in the southwestern United States.

## ST. LOUIS ENCEPHALITIS VIRUS

St. Louis encephalitis virus (SLEV) is a second arbovirus that has caused disease in Arizona since 2015. While the virus was first detected in St. Louis, Missouri, in 1933, the strain currently circulating in the southwestern United States, causing human disease primarily in Arizona, California, and Nevada, likely originated in Argentina (10). In a similar fashion to the WNV study mentioned above, we have partnered with the Coachella Valley Mosquito and Vector Control District and Maricopa County Environmental Services Vector Control Division to determine whether the SLEV is locally maintained or is annually imported to Riverside County, CA, and Maricopa County, AZ. First, our studies have led us to question whether or not this recently imported strain of SLEV is competitively excluded by WNV, as has been suggested for previous strains (11), given geographic proximity and often overlap of the two viruses in positive mosquito pools in Maricopa County. With only 1 of every 100 mosquito pools being positive for WNV, the vector component of the mosquito-avian enzootic cycle does not appear to be consumed such that either virus would be competitively excluded. However, less is known about current susceptibility and immunity of competent avian hosts. Additionally, our preliminary results based on 65 sequenced genomes suggest that SLEV may also be establishing an endemic population in Arizona, alongside WNV, as strains sequenced from 2018 are polyphyletically nested within their 2017 counterparts. While SLEV infection does not result in clinical manifestations as often as WNV infections, in 2015, 23 cases of SLEV were reported in Arizona, and of those, 19 resulted in neuroinvasive disease from which 2 people succumbed (12).

## RABIES

Over the past 3 years, there have been more than 450 wild animal cases of rabies in Arizona (13). During the same time frame, there were 88 human exposures. Although human deaths are infrequent in the United States, public health costs range between $245 and 510 million annually, which includes an estimated 40,000 to 50,000 postexposure prophylaxis treatments (14). These treatments tend to be concentrated in locations where the rabies virus (RABV) has become established in reservoir populations. The Arizona Department of Health Services (ADHS) has been diligently testing, variant typing, and tracking RABV-positive animals and exposed humans throughout the state for several years. We have recently initiated a collaboration with this agency with the overarching goal of characterizing spatiotemporal circulation of the virus. In a similar manner to the SLEV and WNV projects, we will sequence RABV from positive brain stem samples provided by ADHS and overlay the resulting genomes with geographic and timing information. Campaigns to reduce RABV in raccoon populations of the southeastern United States have been successful in locations where oral RABV vaccine baits have been placed (15), and identification of contemporary RABV source populations in Arizona may pave the way for similar intervention campaigns.

## FUTURE DIRECTIONS

We anticipate that these studies, which have been heavily focused on Arizona up to this point, will have major implications regarding the public health-focused utility of incorporating intensely sampled pathogen genomes over local geographic scales for several years. With many state public health labs now gaining access to next-generation sequencing technologies, I anticipate that projects like those that our team has embarked upon will become a mainstay in epidemiological practices. Our hope for each project is that the identification of reemergent circulation patterns will provide proactive intervention opportunities that can reduce pathogen load in the environment.
